# Editorial: The art of reducing futile biomedical research

**DOI:** 10.3389/fmed.2025.1600661

**Published:** 2025-05-12

**Authors:** Ivan Sisa, Ricardo Izurieta, Adrián Llerena, Enrique Teran

**Affiliations:** ^1^Colegio de Ciencias de la Salud, Universidad San Francisco de Quito, Quito, Ecuador; ^2^School of Public Health and Health Sciences, College of Health, Human Services, and Nursing, Carson, CA, United States; ^3^Personalized Medicine and Mental Health Unit, University Institute for Bio-Sanitary Research of Extremadura (INUBE), Badajoz, Spain

**Keywords:** biomedical research, futility, minimization, first class of waste, public health

## Introduction

Biomedical research in its many forms has led to new ways of preventing, diagnosing and treating disease. As a result, people around the world are living longer. For example, in 1900, the average life expectancy of a newborn was 32 years; by 2021, it had jumped to 71 years (1). To achieve this, billions of dollars, millions of people, and an enormous amount of health resources are invested in biomedical research globally each year. Although these endeavors have achieved substantial improvements in the health and wellbeing of individuals and populations, much more could be done if futility in research were avoided/corrected. Historically, futility is often used to indicate that a clinical trial is unlikely to achieve its original goal (2). However, futility can be found in any biomedical research-related activity, and it is translated as waste. For example, it is estimated that 85% of global research investment is wasted each year (3). In 2012, the United States, Canada, Europe, and the Asia-Pacific region invested a total of US$268.4 billion in biomedical research and development (4). This research waste can be categorized into three classes: (i) research that is flawed, poorly conducted, or poorly reported, (ii) research that is incapable of reaching a conclusion different from what is known, and (iii) research that contributes to the imprecision of effect estimates (by adding methodological heterogeneity, underpowered findings, or technological diversion) (5). The present Research Topic, *The art of reducing futile biomedical research*, aims to capture the attention of key stakeholders and provide useful evidence to address this important public health problem, with a particular focus on the first class of waste (e.g., choosing the wrong questions or low priority for research). Therefore, we present four high-quality research articles, including three original contributions and one perspective article.

## Original research

The original research articles in this Research Topic offer valuable insights into how to minimize futile biomedical research efforts using empirical data from Ecuador, England, and France. The first study, “*One hundred years of Ecuadorian biomedical scientific output and its association with the main causes of mortality: a bibliometric study*,” mapped the scientific production in biomedical research in Ecuador and analyzed its association with the main local disease burdens. Among the authors' findings, the predominance of observational studies (79%) was highlighted, along with the fact that private universities are the main producers of biomedical research in the country compared to public institutions, and that 12.5% of the total scientific output (excluding COVID-19-related publications) is dedicated to addressing the main causes of mortality (Sisa et al.). This lack of prioritization of research areas based on national needs contributes to the waste of biomedical research in low- and middle-income countries and has been reported elsewhere (6). The second original study, “*The therapeutic futility paradox: insights from oncological drug litigation in Ecuador*,” analyzed the access to oncological drugs through judicial processes (21 individual claims and 5 collective actions) against the Ecuadorian Ministry of Public Health. Of interest is the striking discrepancy between clinical evidence and judicial decisions. For example, in more than 90% of the judicial processes, the arguments used were improvements in quality of life, overall survival and disease progression; however, according to the U. S. Food and Drug Administration and the European Medicines Agency, only 18.7% of the requested drugs demonstrated such benefits in pivotal clinical trials (Mena Ayala et al.). This significant gap found between scientific evidence and judicial decision-making shows that waste can also be found when relevant, high-quality research is not effectively applied in a clinical/appropriate context (7). The third original article published in this Research Topic, entitled “*Understanding trends in osteoporosis drug prescribing: implications for reducing futile biomedical research*” by Guillemot et al. evaluated trends in osteoporosis drug prescribing in France and England and how medicalization, pharmaceuticalization, and standardization influence the pharmaceutical management of osteoporosis. Their findings show a decline in osteoporosis drug prescribing that may be influenced by changing perceptions of aging, policy influences, and healthcare provider decision-making in France and England. This study adds to the growing body of evidence to reduce futile biomedical research, as understanding prescribing trends would allow efficient resource allocation and improve patient health and quality of life.

## Perspective article

A perspective article in this Research Topic challenged the “publish or perish” paradigm, entitled “*Integrity at stake: confronting “publish or perish” in the developing world and emerging economies*,” the pressure to publish within the academic community has catalyzed unethical practices, including the sale of authorship, the proliferation of paper mills, and the use of artificial intelligence to produce scientific publications, which ultimately jeopardize the credibility and public trust in the scientific community. Therefore, this article provided an overview of how these practices contribute to the futility of biomedical research endeavors. For instance, fraudulent research consumes valuable time of editors, peer reviewers, and journal staff that could otherwise be invested in the analysis of genuine/meaningful submissions (Vasconez-Gonzalez et al.).

## Conclusion

Futile biomedical research occurs regardless of location or income and in a variety of forms. The present Research Topic, thanks to the contributions of all the authors, reviewers, and topic editors involved, shows how answering research questions that are not aligned with national needs, using inappropriate clinical evidence or unethical research practices contributes to the waste in biomedical research. Although this problem is not new and has been in the public eye for some time, its detrimental effects on the progress of science and the wellbeing of people are enormous (7, 8). Paraphrasing the words of John C. Bailar, we can argue that there may be greater danger to the public welfare from scientific dishonesty than from almost any other form of dishonesty (9). We hope that this Research Topic and its valuable findings will inspire further research to address this evolving and timely public health issue.
